# A nationwide population-based cohort study of hospital academic status and survival following colorectal cancer surgery in Finland 1987–2016

**DOI:** 10.1038/s41598-026-38347-4

**Published:** 2026-02-17

**Authors:** Elise Sarjanoja, Kai Klintrup, Pasi Ohtonen, Joonas H. Kauppila

**Affiliations:** 1 Surgery Research Unit, University of Oulu and Oulu University Hospital, Oulu, Finland; 2 Department of Surgery, Länsi-Pohja Central Hospital, Wellbeing Services County of Lapland, Kauppakatu 25, Kemi, 94100 Finland; 3 Upper Gastointestinal Surgery, Department of Molecular Medicine and Surgery, Karolinska Institutet and Karolinska University Hospital, Stockholm, Sweden

**Keywords:** Colorectal carcinoma, Colon, Rectal, Cancer, Surgery, Neoplasm, Survival, Outcome, Academic, Colorectal cancer, Colon cancer, Rectal cancer, Outcomes research, Caecum, Colon, Rectum, Surgical oncology

## Abstract

There is a lack of evidence regarding hospital academic status and survival following colorectal cancer surgery and there is a paucity of data from European countries. The aim of this study was to investigate this association between hospital academic status and mortality after colon and rectal cancer surgery. All 49 032 patients who underwent resection for colorectal cancer in years 1987–2016 in Finland were included, with complete follow-up until December 31, 2019. Primary outcome was all-cause 5-year mortality. Cox regression provided hazard ratios (HR) with 95% confidence intervals (CI) in academic and non-academic hospitals for colorectal surgery, adjusted for calendar period, age, sex, comorbidity, stage, tumor location and oncological therapy. Additionally, colon and rectal cancer surgery were assessed separately. Total colectomies were included in the study, and the cancer location was based on the first location information that was reported by the cancer registry or in the ICD code if not reported in the cancer registry. The manuscript was written according to Equator network guidelines. Compared to academic hospitals, the patients operated in non-academic hospitals had a slightly increased 5-year all-cause mortality (adjusted HR 1.07, 95% CI 1.04–1.11) and also the 30-day and 90-day mortality was increased in patients operated in non-academic hospitals. Sensitivity analysis including only patients operated with confirmed curative intent suggested no differences between academic and non-academic hospitals in colorectal cancer for 5-year all-cause mortality or in 5-year cancer specific mortality, respectively. In analysis including hospital volume as an explanatory covariate, the increase in 5-year mortality in non-academic hospitals compared to academic hospitals was stronger than in main analysis. A pre-planned subgroup-analysis stratified by cancer type (colon, or rectal) suggested a slightly decreased 5-year all-cause mortality in academic institutions for rectal but not for colon cancer surgery. Hospital academic status is associated with slightly improved 5-year mortality in colorectal cancer surgery, but benefits of centralization to academic hospitals may be limited to rectal cancer surgery.

**Supplementary Information:**

The online version contains supplementary material available at 10.1038/s41598-026-38347-4.

## Introduction

Colorectal cancer (CRC) was the third leading cause of cancer death worldwide in 2019^1^. CRC survival is improving in Europe, but there are still persistent significant geographical and age-related disparities according to the EUROCARE-5 study^2^. CRC outcome is heavily dependent upon stage of disease at diagnosis. It typically ranges from a 90% 5-year survival rate for localized stage cancers; 70% for regional cancers; to as low as 10% for people diagnosed for distant metastatic cancer^3^.

The association between increasing annual hospital volume of surgery and long-term survival benefit in colorectal cancer remain unclear^4–9^. While there are several studies to have explored this volume-outcome relationship, including our previous study evaluating colorectal cancer surgeries in Finland in 1987–2016^9^, only few studies comparing survival after colorectal cancer surgery in academic hospitals to non-academic hospitals exist. Academic hospitals are often larger in volume, but also conduct teaching, may have more advanced treatment facilities and options, as well as access to novel or experimental treatments, as compared to non-academic hospitals. A recent American study conducted using the National Cancer Database suggested better survival in metastatic colorectal cancer patients treated in academic hospitals compared to non-academic hospitals^10^. Another study using National Cancer Database suggested no difference between academic and non-academic centers for survival in non-metastatic rectal cancer patients after matching for center annual resection volume^11^. It is unknown whether hospital academic status is associated with long term mortality in colon cancer or in European settings.

The main aim of the present study was to compare survival outcomes for colorectal cancer surgery conducted in academic compared to non-academic hospitals. We hypothesized that hospital teaching status is not associated with colorectal cancer prognosis.

## Methods

### Study design

A Finnish population-based, nationwide retrospective cohort study.

### Cohort

The population of the entire Finland was eligible for this study. The study period covered years 1987 to 2016. All patients with incident colorectal cancer undergoing resectional surgery within a year of cancer diagnosis were identified by using Finnish Cancer Registry (FCR) and Finnish Health Care Register (HILMO). A window of up to one year between the time of cancer diagnosis and surgery was chosen to take diagnostic lag and potential complications in neoadjuvant therapy into account.

### Data sources

Data linkage was based on the immutable personal identity numbers assigned to each resident in Finland. The FCR provided data on all cases of colon and rectum cancer identified using ICD-9 and ICD-10 diagnoses (Supplementary Table 1). We defined right colon as caecum, ascending colon and transverse colon. Left colon was defined as descending colon and sigmoid colon. FCR has been collecting population-based data on cancer incidence since 1953 and after year 1961 sending the information of all cancer cases to the registry has been compulsory. FCR is studied to have a very good quality data for solid tumors^12^. FCR provided information for tumor stage, tumor location, neoadjuvant treatment and curative intent of surgery.

HILMO was used to identify any cancer diagnoses potentially missed by FCR, and to identify all colorectal cancer operations. HILMO collects data for all hospital outpatient visits and ended inpatient admissions and has high positive predictive value for common diagnoses^13^. The operating hospital, date of surgery and operation type were collected from registers by using the classification for Finnish Hospital League 1983 (Sairaalaliiton Toimenpidenimikkeistö 1983) in years 1987–1996 and from year 1997 NOMESCO Classification for Surgical Procedures (Supplementary Table 2). Patient registries provided information on hospital academic status, age at diagnosis, sex, Charlson comorbidity score, and annual volume of surgery. Comorbidity was defined according to the most updated and validated version of Charlson’s Comorbidity Index^14^, including myocardial infarction, congestive heart failure, peripheral vascular disease, dementia, cerebrovascular disease, chronic lung disease, connective tissue disease, ulcer, chronic liver disease, hemiplegia, moderate or severe kidney disease, diabetes, diabetes with complication, tumor, leukemia, lymphoma, moderate or severe liver disease, malignant tumor (excluding colorectal cancer), metastasis, and AIDS. Annual hospital volume was calculated as the number of colon or rectal cancer surgeries performed in each hospital in each year. This number was then assigned to each patient.

The follow-up was based on Death Registry, held by Statistics Finland, providing dates of death until December 31, 2019 and causes of death until December 31, 2018. Cause of death data are highly accurate due to manual checking of all death certificates by a forensic physician^15^ and 100% complete for vital status.

### Exposure

The exposure was the hospital academic status (yes, or no). Academic hospitals were identified using the hospital numbers in the Finnish hospital register, with the five medical university-associated hospitals considered as academic. All the other hospitals were considered non-academic hospitals.

### Outcomes

The primary endpoints were 30-day, 90-day and 5-year all-cause mortality, defined as death by any cause. Secondary endpoints were 5-year cancer-specific mortality, defined as mortality due to colorectal cancer, and 5-year all-cause mortality excluding mortality during the first 90 days during follow up to exclude mortality due to postoperative complications.

### Statistical analysis

Statistical analyses were conducted by an experienced biostatistician (P.O.) according to an a priori study protocol (unpublished). Descriptive statistics of patient and tumor characteristics and outcome indicators were presented in the total group and separately for the exposure groups.

The association between academic and non-academic hospital status and survival were examined using cox regression, providing hazard ratios (HR) and 95% confidence intervals (CI). Follow-up time was calculated from the date of surgery until the date of death, the end of the specified follow-up time, or December 31, 2019 for all-cause mortality (December 31, 2018 for cancer-specific mortality), whichever occurred first. There were four statistical models: (1) crude model without any adjustment, (2) multivariable model adjusted for calendar periods (5-year intervals; 1987–1991, 1992–1996, and so on), age at diagnosis (< 60 years, 60–69 years, 70–79, or ≥ 80 years), sex (male, or female), Charlson comorbidity index (0, 1, or > 1), tumor stage (local, locally advanced, or advanced), tumor location (right colon, left colon, or rectum), and oncological treatment prior to surgery (no, or yes). A sensitivity analysis (model 3) was conducted as in model 2, but including only those patients who were operated with curative intent according to the FCR. Model 4 was conducted as in model 2, but adding annual hospital volume of colorectal cancer surgery as a confounder. Hospital volume was studied in quartiles, the lowest quartile being the reference. Lastly, a stratified analysis by surgery type (colon cancer surgery, or rectal cancer surgery) was conducted for the main outcome variables, including the four models. (Supplementary Table 3). The confounders were selected based on their assumed or literature-based associations with the exposure and the outcome variables, but limited by the availability of variables in used registries.

Due to a proportion of missing tumor stage, multiple imputation for tumor stage was conducted (50 runs), based on the confounding variables. As the point estimates were similar in imputed and non-imputed data, only the results with multiple imputation were presented. All analyses were done using IBM SPSS Statistics version 26 (NY, Armonk).

## Results

### Patient characteristics

There were a total of 74,211 patients with incidental colorectal carcinoma in years 1987 to 2016 in Finland, of whom 49,338 had surgery. Furthermore, 306 patients were operated more than one year apart of first diagnosis, leaving 49,032 patients for analysis.

Out of 49,302 remaining patients 16,752 (34.2%) had surgery in academic and 32,280 (65.8%) in non-academic hospitals. Over time, the number of surgeries increased from 5,413 operations in five-year period in years 1987–1991 to up to 10,945 operations in the last five-year period in years 2012–2016. This increase was mostly driven by increase in surgeries in academic hospitals from 1,395 in 1987–1991 to 4,963 operations in 2012–2016, while the number of surgeries increased in non-academic hospitals from 4,018 to 5,982 operations, respectively (Table 1). Patients operated in non-academic hospitals were older, had more often local stage cancer, and had more often a cancer in right colon (Table 1).

**Table 1:** Clinical characteristics of 49,032 patients undergoing colorectal cancer surgery, stratified by hospital academic status.

Variable	Academic hospital *N* ( %)	Non-academic hospital *N* (%)	Total
Calendar period	16,752	32,280	49,032
1987–1991	1,395 (8.3%)	4,018 (12.4%)	5,413 (11.0%)
1992–1996	1,576 (9.4%)	4,928 (15.3%)	6,504 (13.3%)
1997–2001	2,058 (12.3%)	5,814 (18.0%)	7,872 (16.1%)
2002–2006	2,792 (16.7%)	5,906 (18.3%)	8,698 (17.7%)
2007–2011	3,968 (23.7%)	5,632 (17.4%)	9,600 (19.6%)
2012–2016	4,963 (29.6%)	5,982 (18.5%)	10,945 (22.3%)
Age at diagnosis			
< 60 years	3,736 (22.3%)	5,774 (17.9%)	9,510 (19.4%)
60–69 years	4,836 (29.8%)	8,590 (26.6%)	13,426 (27.4%)
70–79 years	5,192 (31.0%)	11,114 (34.4%)	16,306 (33.3%)
≥ 80 years	2,988 (17.8%)	6,802 (21.1%)	9,790 (20.0%)
Sex			
Male	8,553 (51.1%)	15,968 (49.5%)	24,521
Female	8,199 (48.9%)	16,312 (50.5%)	24,511
Charlson comorbidity index			
0	10,063 (60.1%)	19,799 (61.3%)	29,862 (60.9%)
1	4,182 (25.0%)	7,725 (23.9%)	11,907 (24.3%)
> 1	2,507 (15.0%)	4,756 (14.7%)	7,263 (14.8%)
Tumor stage			
Local	4,248 (25.4%)	9,999 (31.0%)	14,247 (29.1%)
Locally advanced	4,303 (25.7%)	7,083 (21.9%)	11,386 (23.2%)
Advanced	4,574 (27.3%)	8,532 (26.4%)	13,106 (26.7%)
Missing	3,627 (21.7%)	6,666 (20.7%)	10,293 (21.0%)
Tumor location			
Colon	11,195 (66.8%)	23,278 (72.1%)	34,473 (70.3%)
Rectum	5,510 (32.9%)	8,878 (27.5%)	14,388 (29.3%)
Neoadjuvant therapy			
No	13,342 (79.6%)	26,688 (82.7%)	40,030 (81.6%)
Yes	3,410 (20.4%)	5,592 (17.3%)	9,002 (18.4%)
Annual hospital volume of colorectal surgery (median, IQR interquartile range)	128 (89–255)	48 (27–74)	

### Hospital academic status and short-term mortality

The 30-day mortality was higher in non-academic (3.5%) versus academic hospitals (3.0%, HR 1.18, 95% CI 1.03–1.31) in the crude model. In model 2, there was no difference in 30-day mortality comparing non-academic versus academic hospitals (HR 1.02, 95% CI 0.92–1.14) or in the adjusted analysis limited to patients operated with confirmed curative intent (Table 2). However, when annual hospital volume was included in the analysis as a confounder, the mortality in non-academic hospitals was higher compared to academic (HR 1.18, 95% CI 1.06–1.31).

Similarly to 30-day outcomes, the risk of 90-day mortality was increased in patients operated in non-academic hospitals (6.7%) compared to academic hospitals (5.6% HR 1.20, 95% CI 1.11–1.29, Table 2). In model 2, academic status of the hospital was not significantly associated with 90-day mortality (HR 1.04, 95% CI 0.96–1.13, Table 2) or in analysis including confirmed curative intent resections only (Table 2). Again, when annual hospital volume was included in the analysis as a confounder, the mortality in non-academic hospitals was higher compared to academic (HR 1.20, 95% CI 1.11–1.29, Table 2).

**Table 2:** Hospital academic status in relation to mortality outcomes in colorectal cancer patients.

	Number	Academic hospital	Non-academic hospital
30-day all-cause mortality			
Crude	49,032	1 (Reference)	1.18 (1.03–1.31)
Model 2*	49,032	1 (Reference)	1.02 (0.92–1.14)
Model 3†	24,457	1 (Reference)	1.01 (0.84–1.22)
Model 4¥	49,032	1 (Reference)	1.18 (1.06–1.31)
90-day all-cause mortality			
Crude	49,032	1 (Reference)	1.20 (1.11–1.29)
Model 2*	49,032	1 (Reference)	1.04 (0.96–1.13)
Model 3†	24,457	1 (Reference)	1.08 (0.94–1.25)
Model 4¥	49,032	1 (Reference)	1.20 (1.11–1.29)
5-year all-cause mortality			
Crude	49,032	1 (Reference)	1.12 (1.07–1.17)
Model 2*	49,032	1 (Reference)	1.07 (1.04–1.11)
Model 3†	24,457	1 (Reference)	1.04 (0.99–1.08)
Model 4¥	49,032	1 (Reference)	1.18 (1.15–1.22)
5-year cancer-specific mortality			
Crude	49,032	1 (Reference)	1.20 (1.17–1.24)
Model 2*	49,032	1 (Reference)	1.09 (1.06–1.13)
Model 3†	24,457	1 (Reference)	1.05 (0.99–1.10)
Model 4¥	49,032	1 (Reference)	1.20 (1.17–1.24)
5-year overall excluding 90 days			
Crude	45,933	1 (Reference)	1.18 (1.14–1.21)
Model 2*	45,933	1 (Reference)	1.08 (1.04–1.11)
Model 3†	23,539	1 (Reference)	1.03 (0.98–1.08)
Model 4¥	45,933	1 (Reference)	1.18 (1.14–1.21)

*adjusted for confounders calendar period, age, sex, comorbidity, tumor stage, tumor location, and neoadjuvant therapy.

†Adjusted for confounders in Model 2, but only including patients with curative intent.

¥adjusted for confounders in Model 2, but adding annual hospital volume of colorectal cancer surgery as a confounder.

### Hospital academic status and 5-year mortality in colorectal cancer

The five-year all-cause mortality was 40.1% in academic hospitals and 45.6% and in non-academic hospitals (unadjusted HR 1.12, 95% CI 1.07–1.17, Table 2). In model 2, the risk of mortality was still significantly higher in non-academic hospitals (HR 1.07, 95% CI 1.04–1.11, Table 2), but not in patients operated with confirmed curative intent (HR 1.03, 95% CI 0.99–1.08, Table 2). In analysis including hospital volume as an explanatory confounder, the association between non-academic hospitals and 5-year mortality compared to academic hospitals was stronger than in main analysis (HR 1.18, 95% CI 1.15–1.22, Table 2).

### Hospital academic status and secondary outcomes

The 5-year cancer specific mortality was 31.7% in academic hospitals and 36.8% in non-academic hospitals. The patients operated in non-academic hospitals had higher cancer-specific mortality compared to academic hospitals following the results from 5-year all-cause mortality analysis (Table 2). The differences in 5-year all-cause mortality excluding 90-day mortality reflected that of the 5-year all-cause mortality (Table 2).

### Hospital academic status and mortality outcomes in colon cancer

In colon cancer, no differences in 30- or 90-day mortalities were observed between academic and non-academic hospitals (Table 3). The 5-year all-cause, cancer-specific, and 5-year all-cause excluding 90-day mortalities were statistically significantly higher in non-academic compared to academic hospitals in crude model but not in any of the adjusted models, except for 5-year cancer-specific mortality (Table 3). Additional adjustment for hospital volume of colon cancer surgery had no effect on the estimates (Table 3).

**Table 3:** Hospital academic status in relation to colon cancer mortality.

Model	Number	Hospital academic status, colon cancer surgery	
		Academic	Non-academic
30-day all-cause mortality			
Crude	29,668	1 (Reference)	1.04 (0.92–1.18)
Model 2*	29,668	1 (Reference)	0.94 (0.83–1.07)
Model 3†	24,457	1 (Reference)	0.97 (0.77–1.22)
Model 4¥	29,668	1 (Reference)	1.01 (0.85–1.20)
90 day all-cause mortality			
Crude	29,668	1 (Reference)	1.06 (0.97–1.16)
Model 2*	29,668	1 (Reference)	0.96 (0.88–1.06)
Model 3†	24,457	1 (Reference)	1.01 (0.84–1.22)
Model 4¥	29,668	1 (Reference)	1.05 (0.93–1.18)
5 year all-cause mortality			
Crude	29,668	1 (Reference)	1.09 (1.05–1.13)
Model 2*	29,668	1 (Reference)	1.03 (1.00-1.07)
Model 3†	24,457	1 (Reference)	1.02 (0.96–1.09)
Model 4¥	29,668	1 (Reference)	1.04 (0.99–1.09)
5-year cancer-specific mortality			
Crude	29,668	1 (Reference)	1.10 (1.06–1.15)
Model 2*	29,668	1 (Reference)	1.05 (1.01–1.09)
Model 3†	24,457	1 (Reference)	1.04 (0.96–1.11)
Model 4¥	29,668	1 (Reference)	1.05 (0.99–1.11)
5-year overall excluding 90 days			
Crude	27,513	1 (Reference)	1.09 (1.05–1.14)
Model 2*	27,513	1 (Reference)	1.04 (1.00-1.09)
Model 3†	23,539	1 (Reference)	1.02 (0.96–1.09)
Model 4¥	27,513	1 (Reference)	1.04 (0.98–1.09)

*adjusted for confounders calendar period, age, sex, comorbidity, tumor stage, tumor location, and neoadjuvant therapy.

†Adjusted for confounders in Model 2, but only including patients with curative intent.

¥adjusted for confounders in Model 2, but adding annual hospital volume of colorectal cancer surgery as a confounder.

### Hospital academic status and mortality outcomes in rectal cancer

In rectal cancer, the risk of 30-day, 90-day, 5-year all-cause and 5-year cancer specific mortality was statistically significantly higher in non-academic hospitals compared to academic hospitals, and this effect remained after adjustment for confounders (Table 4). In analysis restricted to patients with confirmed curative intent, this effect was mitigated. In models adjusted for annual volume of rectal cancer surgery, academic hospitals had lower short- and long-term mortality than non-academic hospitals.

**Table 4:** Hospital academic status in relation to rectal cancer mortality.

Model	Number	Hospital academic status, rectal cancer surgery	
		Academic	Non-academic
30-day all-cause mortality			
Crude	19,364	1 (Reference)	1.49 (1.23–1.81)
Model 2*	19,364	1 (Reference)	1.23 (1.01–1.50)
Model 3†	10,288	1 (Reference)	1.10 (0.82–1.49)
Model 4¥	19,364	1 (Reference)	1.34 (1.04-1,71)
90 day all-cause mortality			
Crude	19,364	1 (Reference)	1.49 (1.30–1.72)
Model 2*	19,364	1 (Reference)	1.23 (1.07–1.43)
Model 3†	10,288	1 (Reference)	1.22 (0.97–1.55)
Model 4¥	19,364	1 (Reference)	1.30 (1.08–1.56)
5 year all-cause mortality			
Crude	19,364	1 (Reference)	1.32 (1.26–1.38)
Model 2*	19,364	1 (Reference)	1.13 (1.08–1.19)
Model 3†	10,288	1 (Reference)	1.05 (0.98–1.12)
Model 4¥	19,364	1 (Reference)	1.10 (1.03–1.17)
5-year cancer-specific mortality			
Crude	19,364	1 (Reference)	1.37 (1.30–1.44)
Model 2*	19,364	1 (Reference)	1.16 (1.10–1.23)
Model 3†	10,288	1 (Reference)	1.06 (0.98–1.14)
Model 4¥	19,364	1 (Reference)	1.09 (1.02–1.17)
5-year overall excluding 90 days			
Crude	18,420	1 (Reference)	1.30 (1.24–1.37)
Model 2*	18,420	1 (Reference)	1.13 (1.07–1.18)
Model 3†	9,927	1 (Reference)	1.04 (0.97–1.12)
Model 4¥	18,420	1 (Reference)	1.08 (1.01–1.15)

*adjusted for confounders calendar period, age, sex, comorbidity, tumor stage, tumor location, and neoadjuvant therapy.

†Adjusted for confounders in Model 2, but only including patients with curative intent.

¥adjusted for confounders in Model 2, but adding annual hospital volume of colorectal cancer surgery as a confounder.

## Discussion

The results of this population-based, nationwide cohort study suggest increased mortality in colorectal cancer patients operated in non-academic hospitals compared to academic hospitals. However, the increased mortality is limited to rectal cancer only, which cannot be explained solely by higher volume of surgery in academic hospitals.

The population-based design and virtually complete inclusion of all patients with colorectal cancer surgery in the whole Finland are the main strengths of this study. Validated nationwide registers enabled retrieval of accurate information about the exposures, outcomes, and relevant covariates. The reliable identification of all eligible patients and the complete follow-up was permitted by the unique personal identity numbers and high-quality registries. Confounding bias remains a limitation in observational studies. This was mitigated by adjusting for several potential confounders, although residual confounding cannot be ruled out. The long inclusion period of three decades might be considered a weakness. The patients operated during the latest study period still had a minimum of 2 years of follow up for disease-specific mortality and 3 years for all-cause mortality. However, they were only a minor part of the whole cohort, so this limitation only reduces the statistical power, and does not impact the risk estimates. The care for colorectal cancer patients has been greatly improved in the past three decades. Better perioperative care, oncological treatment strategies introducing neoadjuvant and adjuvant treatment have impacted the long-term survival. Furthermore, minimally invasive surgery has become an integral part of CRC management. The lack of specific data on oncological treatments and unidentified role of emergency surgery can be considered as weaknesses in this study.

However, the calendar periods lasting 5 years were adjusted for in the analyses, which mitigates this confounding to some extent. Only the crude staging by the cancer registry was available, based on descriptive stages, namely local, locally advanced, and advanced stage in FCR and therefore we could not use *Union for International Cancer Control (UICC) stage of cancer.* That can be considered as a weakness in this study.

Registration of stage and oncological treatments are not 100% complete in the FCR and suffer of some non-differential misclassification due to underestimating the proportion of advanced cancers and patients receiving oncological treatments. Defining curative therapy is based on FCR records and many patients undergoing curative-intended therapy, especially those with rectal cancer, might have been left out from these sub-analyses, leading to lower power but should not cause major changes in the point estimates. There was some missing data on tumor stage, which was handled using multiple imputation. Lack of complication data can be considered as a weakness in this study.

In a US study with metastatic colorectal carcinoma the survival was better in patients treated in academic institutions. The patients treated in academic hospital had more radical resections of liver and lung metastases and had more often neoadjuvant treatment^10^. Another recent study using National Cancer Database suggested no difference between academic and non-academic centers for survival in non-metastatic rectal cancer patients after matching for center annual resection volume^11^. In the current study colorectal cancer 5-year mortality in academic hospitals was lower than in non-academic hospitals. The long-term survival benefit was more apparent in patients with rectal cancer, while in colon cancer the differences between academic and non-academic hospitals was not significant except for 5-year cancer-specific mortality.

The findings could be explained by a number of factors. In higher volume hospitals or in academic hospitals surgeons may have specialized in doing colorectal operations more often and with higher volume than in non-academic hospitals. Multidisciplinary treatment pathways and perioperative practices may explain some of the differences. Complications are more common in rectal cancer surgery than colon cancer surgery. In severe complications the access to more advanced resources available in the academic centers could therefore reduce failure to rescue compared to non-academic centers, than in the context of colon cancer. No information on individual surgeons or their surgical volume was available, and therefore this could not be assessed in the current study.

## Conclusion

According to the results of this study, hospital academic status is associated with improved colorectal cancer survival. Subgroup analysis indicates that this improved survival could be limited to rectal cancer, and not colon cancer. Further large studies in different types of healthcare systems are required to fully evaluate the impact of hospital academic status on survival after colorectal cancer surgery.

## Supplementary Information

Below is the link to the electronic supplementary material.

Supplementary Material 1

## Data Availability

The data used in this study are available from Findata, but restrictions apply to the availability of these data, which were used under license for the current study, and thus not publicly available. It is recommended to contact the corresponding author for further information.
