# Supplementary material for: A nationwide population-based cohort study of hospital academic status and survival following colorectal cancer surgery in Finland 1987–2016
Source: Sci Rep. 2026 Feb 17;16:9478. doi: 10.1038/s41598-026-38347-4 (PMC13004910; doi:10.1038/s41598-026-38347-4)
Supplement: Supplementary file 1 — Supplementary Material 1 [file 41598_2026_38347_MOESM1_ESM.pdf]

Supplementary table 1: ICD-codes of colon and rectum carcinoma.

	ICD-10	***ICD-9***
Malignant neoplasm of cecum	C18.0	1534
Malignant neoplasm of ascending colon	C18.2	1536
Malignant neoplasma hepatic flexure	C18.3	1530
Malignant neoplasm of transverse colon	C18.4	1531
Malignant neoplasm of splenic flexure	C18.5	1537
Malignant neoplasm of descending colon	C18.6	1532
Malignant neoplasm of sigmoid colon	C18.7	1533
Malignant neoplasm of overlapping sites of colon	C18.8	1538
Malignant neoplasm of colon, unspecified	C18.9	1539
Malignant neoplasm of rectosigmoid junction	C19	1540
Malignant neoplasm of rectum	C20.	1541
Malignant neoplasm of rectum, unspecified or unknown		1548

Supplementary table 2: Finnish surgical codes for colon and rectum resections:

	NOMESCO open	NOMESCO laparoscopic	NOMESCO endoscopic	Finnish Surgical Codes (1983)
Colon resections JFB				
Ileocaecal resection	JFB 20	JFB 21		6403
Right hemicolectomy	JFB 30	JFB 31		6404
Other resection comprising small intestine and colon	JFB 33	JFB 34		
Resection of transverse colon	JFB 40	JFB 41		
Left hemicolectomy	JFB 43	JFB 44		6405
Other resection of colon	JFB 50	JFB 51		6406
Total colectomies JFH				
Total colectomy and ileorectal anastomosis	JFH 00	JFH 01		6407
Total colectomy and ileostomy	JFH 10	JFH 11		6408
Proctocolectomy and ileostomy	JFH 20			6410
Total colectomy, mucosal proctectomy and ileoanal anastomosis without ileostomy	JFH 30			
Total colectomy, mucosal proctectomy, ileoanal anastomosis and ileostomy	JFH 33			

Proctocolectomy and continent ileostomy	JFH 40			
Other total colectomy	JFH 96			
Other operation in this group				6419
Operations of rectosigmoideum				
Anterior resection (sigmoid colon and rectum)				6434
Abdominoperineal resection (APR) and colostomy				6435
Transrectal resection				6436
Other operation in this category				6449
Operations of sigmoid colon JFB				
Resection of sigmoid colon	JFB 46	JFB 47		
Resection of sigmoid colon with partial proctectomy	JFB53	JFB 54		
Resection of sigmoid colon with end colostomy	JFB 60	JFB 61		
Other resection of colon with proximal colostomy and closure of distal stump	JFB 63	JFB 64		
Other partial excision of intestine	JFB 96	JFB 97		
Operations of rectum JGA and JGB				

Proctotomy and excision of lesion of rectum	JGA70			
Transanal excision of lesion of rectum	JGA 73		JGA 75	
Other proctotomy or local operation of rectum	JGA 96	JGA 97	JGA 98	
Partial proctectomy and colorectal or coloanal anastomosis	JGB 00	JGB 01		
Partial proctectomy with partial excision of mesorectum	JGB 03	JGB 04		
Partial proctectomy with total excision of mesorectum	JGB 06	JGB 07		
Partial proctectomy and end colostomy	JGB 10	JGB 11		
Abdominoperineal excision of rectum	JGB 30			
Laparoscopic and perineal excision of rectum		JGB 31		
Abdominoperineal excision of rectum with intersphincteric dissection	JGB 33	JGB 34		
Wide excision of rectum	JGB 36			

Supplementary table 3: Models used in this study

Adjustments	Crude model without any adjustment	Model 2 multivariable model	Model 3	Model 4	Stratified analysis by surgery type (colon cancer surgery, or rectal cancer surgery
calendar period		x	x	x	x
age		x	x	x	x
sex		x	x	x	x
Charlson comorbidity index		x	x	x	x
tumor stage		x	x	x	x
tumor location		x	x	x	x
neoadjuvant therapy		x	x	x	x
operated with curative intent			x		x
annual hospital volume				x	x
surgery type (colon, rectum)					x
